# Case Studies of a Simulation Workflow to Improve Bone Healing Assessment in Impending Non-Unions

**DOI:** 10.3390/jcm13133922

**Published:** 2024-07-04

**Authors:** Tanja C. Maisenbacher, Saskia Libicher, Felix Erne, Maximilian M. Menger, Marie K. Reumann, Yannick Schindler, Frank Niemeyer, Lucas Engelhardt, Tina Histing, Benedikt J. Braun

**Affiliations:** 1 Department of Trauma and Reconstructive Surgery, Eberhard Karls University Tuebingen, BG Klinik Tuebingen, 72076 Tuebingen, Germany; tmaisenbacher@bgu-tuebingen.de (T.C.M.); slibicher@bgu-tuebingen.de (S.L.); ferne@bgu-tuebingen.de (F.E.); mmenger@bgu-tuebingen.de (M.M.M.); mreumann@bgu-tuebingen.de (M.K.R.); thisting@bgu-tuebingen.de (T.H.); 2 Project Team OSORA—Medical Fracture Analytics, Ulm University, Helmholtzstr. 20, 89081 Ulm, Germany; yannick.schindler@osora.de (Y.S.); frank.niemeyer@osora.de (F.N.); publication@lucasengelhardt.de (L.E.)

**Keywords:** fracture healing, non-union, mal-union, individualized, simulation, vascularization, biomechanics

## Abstract

**Background:** The healing potential of a fracture is determined by mechanical and biological factors. Simulation-based workflows can help assess these factors to assist in predicting non-unions. The aim of this study was the introduction of two use cases for a novel patient-specific simulation workflow based on clinically available information. **Methods:** The used software is an extension of the “Ulm Bone Healing model” and was applied in two cases with non-union development after fracture fixation to show its principal feasibility. The clinical and radiographic information, starting from initial treatment, were used to feed the simulation process. **Results:** The simulation predicted non-union development and axial deviation in a mechanically driven non-union. In the case of a biological non-union, a slow, incomplete healing course was correctly identified. However, the time offset in callus bridging was discordant between the simulation and the distinctly slower healing response in the clinical case. **Conclusions**: The simulation workflow presented in the two clinical use cases allowed for the identification of fractures at risk for impending non-union immediately after the initial fixation based on available clinical and radiographic information. Further validation in a large non-union cohort is needed to increase the model’s precision, especially in biologically challenging cases, and show its validity as a screening instrument.

## 1. Introduction

Despite substantial research and progress concerning fracture treatment and healing, non-union still occurs in about 5–10% of all fracture cases [1]. Depending on the fracture location and surrounding conditions, the rates can increase to up to 20% [2]. Major risk factors include open fractures and high degrees of soft tissue injury in terms of biological factors, and stability as well as the osteosynthetic construct setup, in terms of mechanical factors [3,4,5,6]. Due to long healing periods, non-union treatment not only poses a substantial socioeconomic impact on healthcare systems but also has a significant effect on health-related quality of life concerning both physical and mental health [7,8]. The diagnosis of fracture non-union is commonly a multistage process: Pain and loss of function are the first clinical symptoms driving further analysis. Radiological diagnostics by X-ray and computed tomography (CT) confirm failure or delay of fracture healing. Deciding on when a non-union is established and needs further revision requires a high degree of expertise and is subject to the timing associated with diagnostics [9]. There is still no score or gold standard guidelines universally agreed upon concerning the diagnosis of a non-union, further complicating its treatment and timing in clinical practice [10,11,12,13,14]. In particular, in the early phases of healing, adequate fracture healing monitoring is challenging [15]. Thus, identifying a non-union adequately often takes up to 6–9 months, potentially delaying the initiation of revision therapy [2].

Fracture healing after fixation is driven by both mechanical and vascularity-associated factors. Mechanical conditions in the fracture gap depend on the type of osteosynthesis, the fracture morphology, and patient loading [16]. Claes et al. defined experimental healing boundary conditions and hypothesized two major mechanical influences: relative strain and hydrostatic pressure [17]. Both underly and influence the dynamics of the bone healing process. Secondary bone healing is characterized by different stages [18]. The inflammation stage is characterized by hematoma and the infiltration of different cell types and growth factors like bone morphogenetic proteins. The formation of a soft callus by endochondral ossification and replacement by a hard callus afterward is followed by the final stage of bone healing and bone remodeling, leading to the recovery of the lamellar bone to the state before the fracture. Stiffness and loading define interfragmentary strain and thus cell differentiation. Additionally, adequate vascularization is essential to supply the fracture site with sufficient oxygenation and growth factors [19]. Insufficient blood supply can be related to patient risk factors such as diabetes and nicotine abuse, as well as injury- and surgery-related factors, such as open fractures or the surgical approach [2].

In principle, numerical simulations can predict and visualize the fracture healing process, and these techniques have already been applied to limited case series in clinical settings. Their main limitation is the degree of precision given in their healing assessment, largely influenced by the number of input parameters used for the simulation [20]. Combining motion-capturing data to define individual loading and radiological data, it was possible to simulate the mechanical fracture environment in patients with tibial non-union [21]. By mapping interfragmentary strain and the von Mises stress distribution within different points of the fracture gap, the healing potential of a lower leg fracture situation can be predicted based on individual loading characteristics, gait speeds, and fracture geometry [21,22]. The principal feasibility of this technique has since been shown in other fracture and non-union situations both in the upper and lower extremity [2]. Many of these models are driven by motion capturing and are aimed at either mechanics or biology. Based on the Ulm Bone Healing model, software was developed that allowed for the simulation of the healing process of different fracture situations using both mechanical and clinical input, taken only from clinical imaging and history [23,24]. Thus far, this model has been used to simulate the healing process in various experimental scenarios in sheep, as well as in the human tibia and the distal radius. Recently, the algorithm was applied to human femoral shaft fractures, where it was able to accurately predict the healing potential and clinical course [25]. While the principal detection capability of the simulation workflow in femoral fractures, including non-union cases, was shown, a further more detailed analysis of the concept’s feasibility in dedicated, challenging healing situations is needed [25].

The aim of the present study was to introduce two use cases of an adapted simulation workflow based on the Ulm Bone Healing model in impending non-unions of the lower extremity and highlight its strengths and weaknesses to detect healing disturbance both in mechanically and biologically challenging cases. 

## 2. Materials and Methods

Principal ethical approval was obtained from the ethical committee of Tuebingen University (application number 318/2022BO2, 840/2019B02, 13 July 2022). Informed consent including the use of patient data for analysis and publication, anonymization and secure data storage, the potential data safety risks, and patients’ right to withdraw consent was given by both patients to participate in the collection of movement data and the fracture and non-union simulation based on their clinical and imaging data. As a feasibility study, we aimed to highlight the workflow’s general applicability in two challenging cases, one mechanically and one biologically driven, to provide a foundation for emerging systematic research. Both patients were consecutively enrolled after the availability of the simulation workflow at our center. Additional consent was given to share the data with the simulation provider in a pseudonymized fashion over a DSGVO conform server. We followed the Danish Orthopaedic Trauma Society’s definition of a non-union for this article, where a fracture that will not heal without further intervention is considered a non-union [11]. A fracture that is delayed beyond the expected healing course is considered a delayed union.

### 2.1. Case Data

**Figure 1:**
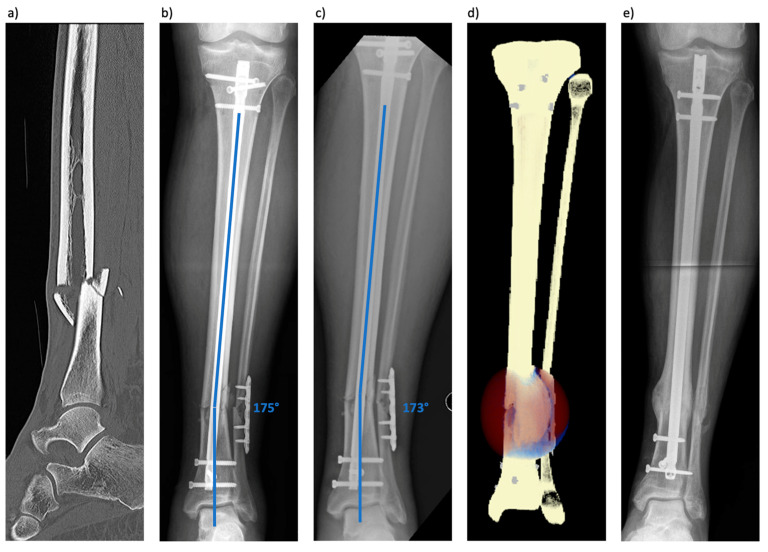
(**a**) Lateral CT image of the lower leg fracture (AO/OTA 42B3c). (**b**) Ap (anteroposterior) radiograph taken one day after initial surgery. The tibial fracture was addressed by nailing and the fibula fracture by plate osteosynthesis. (**c**) Ap radiograph taken 3 weeks after the initial surgery showed progressive loss of reduction and no signs of healing. (**d**) The simulation results at the final timepoint with secondary dislocation in accordance with the clinical result and tissue differentiation is shown for the 100% body weight weight-bearing scenario (brown-red = connective tissue, blue = cartilage, light orange = woven bone, light yellow = lamellar bone). (**e**) Ap radiograph taken about 6 months post-revision surgery showing timely consolidation and correction of leg axis.

The first case is a 54-year-old healthy patient suffering from a closed tibial shaft fracture (AO/OTA 42B3c) and a distal fibular fracture (Figure 1) after being hit by a tree. The closed lower leg fracture was immediately addressed by internal nail fixation of the tibia and an open reduction procedure of the distal fibula with plate osteosynthesis at an external institution. Two months later, the patient was admitted to our clinic due to ongoing pain and increasing axial deviation. The CT scan showed an axial deviation of the tibia and fibula. A two-staged revision was performed due to the slow healing of the fibular-sided incision. The wound was excised, and the plate was removed during the first step. No bacterial contamination was detected. One week later, exchange nailing with reaming was performed. The patient was changed from a 9 mm to a 10 mm diameter nail. Axis deviation was corrected with percutaneously placed blocking screws distally both in ap and mediolateral directions. Postoperatively, the patient was immediately prescribed full weight-bearing. Seven months later, complete fracture healing was documented (Figure 1).

**Figure 2:**
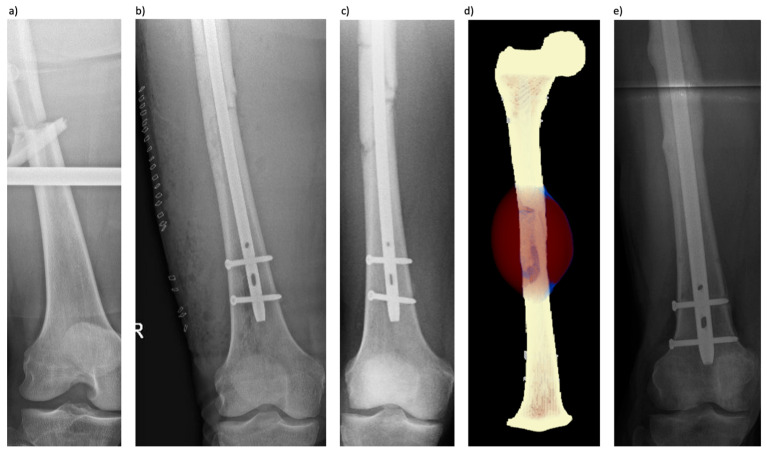
(**a**) Ap radiograph of the dislocated femur fracture (AO/OTA 32B2b). (**b**) Ap radiograph showing the initial nail osteosynthesis one day after surgery with the large lateral-based incision used for the open reduction procedure of the intercalary fragment. (**c**) Four months after trauma, no consolidation or callus formation was observed in the radiograph. (**d**) The simulation results at the final timepoint with tissue differentiation are shown for the 5% body weight weight-bearing scenario (brown-red = connective tissue, blue = cartilage, light orange = woven bone, light yellow = lamellar bone). (**e**) Ap radiograph taken about 5 months post-revision surgery showing timely consolidation.

The second case is a 55-year-old patient with a closed femoral shaft fracture (AO/OTA 32B2b) (Figure 2). The patient suffered from cardiovascular disease with previous stent implantation, high blood pressure, and gastroesophageal reflux disease. His regularly taken medication was aspirin, bisoprolol, and rosuvastatin. He was treated at an external institution with antegrade femoral nailing. An additional lateral incision was performed to openly reduce an interposed fracture fragment, and the fracture hematoma was rinsed out as per the surgical report. Five months after the initial treatment, the patient came to our clinic due to ongoing pain at the former fracture site and persisting inability to bear weight on the extremity. The CT scan showed delayed fracture healing with no callus formation and visible fracture gaps. Based on the history and clinical imaging, the non-union was deemed to be primarily of biological cause, so 3 weeks later, the patient underwent revision surgery with reamed exchange nailing. The patient was changed from a 9 mm to a 12 mm diameter nail. Immediate full weight-bearing was prescribed postoperatively. Seven months later, the patient declared to have no more pain, and the conventional radiograph, as well as the CT scan, showed sufficient callus formation and fracture healing (Figure 2).

### 2.2. Digital Twin of the Fracture Situation

The Ulm tissue-level bone healing model predicts the evolution of different tissues, woven and lamellar bone, fibrocartilage, and fibrous connective tissue, throughout fracture healing [23,24,26]. Both mechanical and biological stimuli are taken into consideration. The simulation is based on established biomechanical observations and the mechano-regulating hypothesis by Claes and Heigele [27,28]. Distortional and dilatational strains have been identified to be determining mechanical factors for tissue differentiation and remodeling [26]. In addition, biological parameters like the physiological condition of the tissue surrounding the fracture and vascularization were considered in the simulation by using fuzzy logic rules. The current strain at every point of the healing process was analyzed by the finite element method (Ansys^®^ Mechanical APDL, Version 2020 R2; Ansys, Inc., Canonsburg, PA, USA). The presented model was therefore able to predict the development of the different tissue types over time and the time till fracture consolidation. The simulation of bone healing was applied for 240 days, and the simulated healing process was then compared to the original outcome. The time of consolidation was derived automatically from the primary simulation results using a path-search algorithm to detect connections of lamellar bone in all four quadrants of the bone. If the simulation predicted bony bridging in at least three quadrants within the simulated days, the case was classified as “union”; otherwise, the case was classified as a “non-union”. A detailed description of the general simulation workflow can be found in recent publications [23,24], and it was clinically applied to femoral fractures in the study by Degenhart et al. [25].

### 2.3. Application to the Clinical Cases

To simulate the healing process, the first step was to create a finite element model of the individual bone anatomy, fracture, and osteosynthesis of the patient based on the provided CT scans in order to reconstruct the postoperative situation. For each of the cases, 3D Slicer [29] was used to segment the tibia/fibula or the femur, respectively, as well as the implant from the CT image data. This step enabled the generation of an accurate three-dimensional (3D) geometric representation of the fractured bone. Since the CT scans were not taken immediately after the surgery but at a later time, we manually realigned the fragments based on the postoperative X-ray scans. 

The fracture area was augmented with a so-called “healing domain” that encompassed the entire fractured area, representing the area in the immediate vicinity of the fracture where we expected tissue formation/differentiation due to mechanical and biological stimuli to take place, according to our tissue differentiation algorithm. The geometries were discretized into finite elements using appropriate meshing techniques (Ansys^®^ Mechanical APDL, Version 2020 R2; Ansys, Inc., Canonsburg, PA, USA). For the initial tissue composition, we assumed that the cortical and cancellous bone consisted of 100% lamellar fully vascularized bone, while the remaining tissue (in between and proximally to the fracture) was assumed to consist of initially avascular soft tissue. 

To determine the loading conditions, the relevant literature on physiological loading scenarios for femoral and tibia fractures was consulted. These loading conditions were then applied to the FE model to simulate the mechanical response of the fractured bone. In both cases, the patients were assumed to bear full weight after surgery. According to our recent study with femur fractures [25], the maximum load occurring during the normal gait cycle was assumed. Therefore, the muscle and joint loading conditions, as stated by Heller et al. (2005) [30], were used, which allowed us to express loading in terms of the percentage of patients’ body weight. The tibia case was loaded as described by Zhao et al. by applying a 55/45% split of the peak load during the gait cycle (2.2 times the body weight) on the medial and lateral compartments of the tibial plateau [31].

Using the reconstructed geometry and the patient’s weight, the simulations were able to be performed within 24 h of simulation time on a high-performance workstation (AMD Ryzen 5900X, 128 GB RAM, AMD, Santa Clara, CA, USA). See Supplementary Materials for more details.

## 3. Results

### 3.1. Patient 1 (Tibial Fracture)

**Figure 3:**
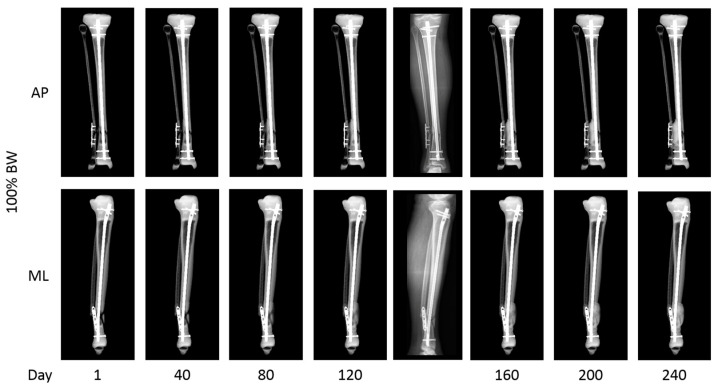
Fracture healing simulation assuming the full weight-bearing of the patient. The simulation shows the failure of the union of the fracture with progressive axial deviation. Simulated radiographs are shown for selected days after the initial surgery (1, 40, 80, 120, 160, 200, and 240 days after surgery) in an ap (**top row**) and lateral projection (ML, mediolateral) (**bottom row**). After day 120, a clinically matched actual lower leg radiograph before the revision is shown.

The fracture healing simulation for this patient revealed that this fracture was not going to unite. During the 240 simulation days, cortical bridging was never seen for more than two cortices (Figure 3). Additionally, the simulation showed a deviation of the fibular axis throughout the healing process, in line with the clinical presentation of an increasing leg axis deviation before revision.

### 3.2. Patient 2 (Femoral Fracture)

**Figure 4:**
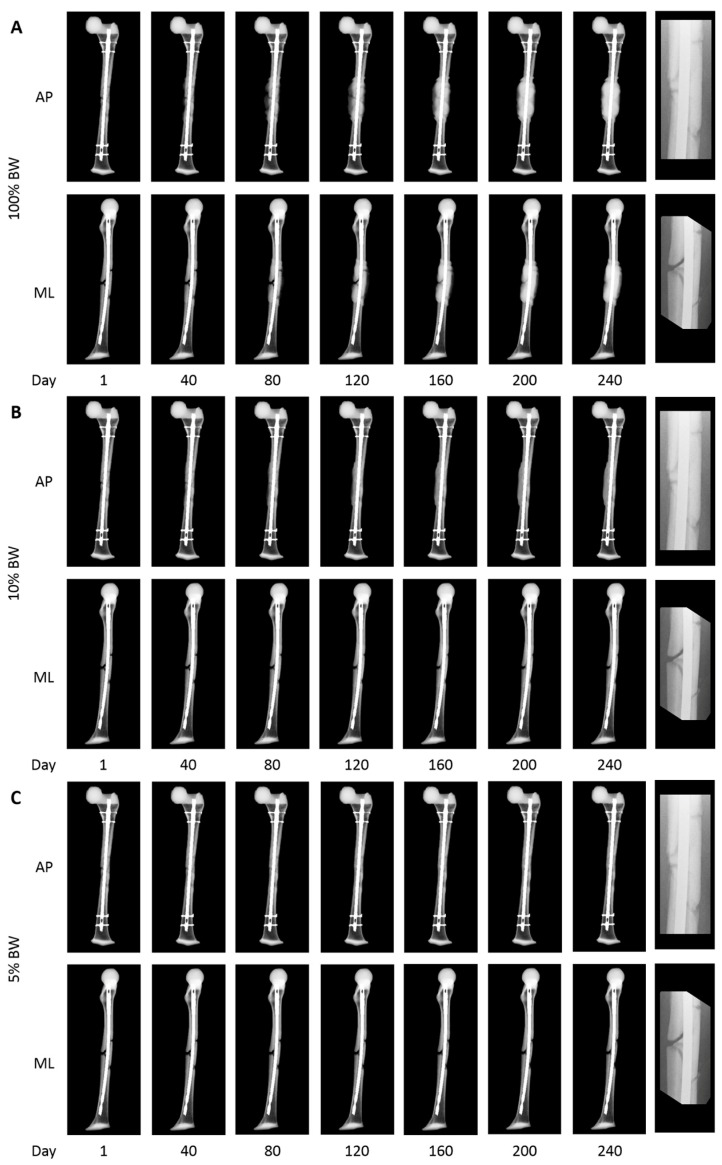
(**A**) Simulation of fracture healing according to full weight-bearing during 240 days. This simulation shows a slow fracture healing with cortical bridging of 3 out of 4 cortices after 184 days. (**B**) This simulation shows the fracture healing assuming a weight-bearing state of 10% of body weight. Here, no consolidation could be observed, and less callus formation occurred. (**C**) Simulation of fracture healing assuming a weight-bearing state of 5% of body weight. Again, no fracture healing is shown with a continuously atrophic intercalary fracture fragment. After day 240, for each series, a clinically matched actual radiograph focused on the callus situation before the revision is shown.

Assuming an immediate full weight-bearing state, the fracture healing simulation for this patient concluded that this fracture was going to unite. During the 240 simulation days, cortical bridging was seen in this patient for three out of the four cortices after 184 days. The anterior cortex did not show bone healing within this period (Figure 4). Calculations with lower levels of weight-bearing in the region of 5–10% of the patient’s body weight, in line with the clinical situation, resulted in the correct prediction of non-union development with the progressive dissipation of the interposed fragment (Figure 4).

## 4. Discussion

The present study shows two use cases of a simulation workflow based on the Ulm Bone Healing Model to analyze the healing course of challenging fractures progressing to non-unions. The model was able to determine the impending non-union in a mechanically challenging fracture case and also the increasing axial deviation that occurred during the initial treatment aftercare. Furthermore, the simulation of a mainly biologically driven non-union identified its slow healing course while also highlighting the system’s limitations pertaining to individual patient weight-bearing. The current work shows the opportunities as well as pitfalls associated with this technique.

Determining the healing potential of fractures, especially during the early treatment course, remains challenging under clinical conditions, thus often leading to a significant delay in the diagnosis of a fracture non-union [2]. In particular, the biological viability of the patient and fracture situation is difficult to assess in all detail, as well as the interaction of mechanics, biology, and the individual situation of each patient according to, e.g., surgical treatment, and compliance. Several clinical scores have aimed at determining the risk for non-union early during the treatment of a lower extremity fracture, most prominently the Non-Union Risk Development Score [32]. While this score gives an estimation of the risk of healing delay, it only incorporates simple clinical information, without fracture mechanics or osteosynthetic construct, thus leaving a degree of imprecision, which makes it more of a general risk assessment tool to identify patients at higher risk for non-union. Chloros and colleagues have recently looked at the four most common clinical scoring systems, namely the Leeds–Genoa Non-Union Index (LEG-NUI), the NURD, the FRACTING score, and the Tibial Fracture Healing Score (TFHS) [33]. They have shown high positive and negative predictive values, especially for the LEG-NUI score, in a retrospective application to tibial non-union cases. An advantage of these scoring systems is that they can be applied during the first 6–12 weeks of clinical treatment and are based on clinical information. As part of their conclusion, the authors advise that further prospective studies are needed to provide more evidence regarding the scores’ useability and predictability. Another clinically applicable prediction method to determine the fracture healing potential is radiographically [34]. Different scores have been described for both the upper and lower extremity most prominently for tibial (Radiographic Union Score Tibia (RUST) and modified RUST (mRUST)), femoral (Radiographic Union Score Hip (RUSH)) and humerus (Radiographic Union Score Humerus (RUSHU)). While these scores greatly improve interrater reliability, only limited evidence exists regarding cut-off values and their prospective use. 

In an attempt to increase the level of detail in assessing the healing potential of a fracture situation, different simulation models have been established and used in clinical settings. Dailey and colleagues have described a simulation approach based on low-dose CT imaging data that were used for virtual mechanical testing and provided a structural callus assessment to predict time to union and enable the early diagnosis of compromised healing, as early as 12 weeks after a fracture [35]. Other research groups have described further healing simulations based on motion capturing and ground reaction data in conjunction with patient imaging to determine the interfragmentary strain of fractures and non-unions [22]. They were able to determine the influence of different mechanical parameters, such as weight-bearing, gait speed, and the overall setup of the osteosynthetic construct, on the healing chances of a fracture. However, these simulations are based on additional information that goes beyond routinely available clinical data and primarily focus on fracture mechanics. Few simulations in a clinical situation have already employed a combined biological and mechanical simulation approach [23,24,25]. 

The results of the simulation of patient 1 highlight the system’s principal capability to assess the mechanical situation based solely on available clinical information. It was able to determine that this fracture would not heal and that the leg axis deviation, which was also encountered during the clinical course, would increase. The precision and capability of a mechanical-based fracture simulation in non-union cases to determine the risk of implant failure and healing delay have already been reported by other groups [22]. However, many of these systems are based on additional information and sensors, such as inertial measurement units or the ground reaction force, limiting their general clinical applicability. The workflow presented here is based only on clinical and imaging information obtained during standard-of-care treatment without additional measures. 

The results of the second patient highlight both the opportunities and shortcomings of the presented workflow in a challenging biological situation. The mechanical setup of the initial treatment was sound with good reduction and intramedullary nail osteosynthesis with good sufficient nail fit, well within accepted ranges [36]. Accordingly, the mechanics during the simulation were seen as adequate. Moreover, assuming immediate full weight-bearing, in this case, would have led to the algorithm assuming a slow but complete fracture healing, with cortical bridging on three sides after a little over 180 days. This did completely change in weight-bearing simulation scenarios equivalent to 5–10% body weight. Here, no healing within the simulation timeframe of 240 days was seen in accordance with the clinical course. The patient’s fracture did not exhibit adequate healing response at 5 months with severely limited function and mobility due to continuing pain at the non-union site. At the request of the patient, the decision was made to revise the non-union with an exchange nailing procedure, and subsequent fracture healing was observed. So, despite the very slow healing course predicted in a full weight-bearing scenario, a different healing outcome was obtained clinically. Only significantly adapting the weight-bearing condition led to the correct prediction. This outlines the need for adequate monitoring of the patient and adapting the simulation to the patient’s individual capability of weight-bearing and again shows the system’s capability for the correct prediction of the mechanical situation without the need for additional data apart from routine control imaging. If full weight-bearing is not possible, a different mechanical impact on the fracture site has to be assumed. Understandably, the simulation can only assess the biological parameters included in the available clinical input data. Without knowing the surgical report of the initial treatment performed at an outside institution, the real extent of surgical tissue damage cannot be identified by clinical and imaging data alone. During the treatment, the large intercalary fragment was completely removed, reinserted, and openly reduced during nail passage, potentially devascularizing large portions of the fracture situation. The system only considers clinical information and imaging, not taking into account the patient’s decreased biological state due to the additional surgical trauma. Future versions of the simulation can certainly be adapted to incorporate these biological healing challenges in more detail. 

As opposed to other simulation techniques, in fracture non-union, mainly focusing on individual fracture mechanics, the current approach also incorporates biological aspects through routinely available data. This underlines the system’s potentially broad applicability in all clinical settings and beyond specialized study centers and lab situations. The information for the clinical course and the risk for delayed healing can be assessed immediately after the initial fracture treatment, allowing for the identification of patients at risk for delayed healing who require a closer observation early on. The time taken for the simulation algorithm is clinically feasible and took about 48 h per case for the current analysis. As the simulation is based on readily available clinical information, the main cost driver is the cost of personnel to set up and run the simulation. This is certainly a limitation compared to clinical scoring systems with comparable early applicability and without the associated cost. Future hardware and software developments will likely increase the speed and precision of the simulation workflow significantly, further increasing its clinical usefulness. Integration into the clinical information system could help provide a simulation with minimal a number of physician and patient input. Certainly, further studies are needed to refine the workflow’s detection capability and predictive precision.

Provided further validation of the workflow shows its continued precision along the lines of the results presented here, the earlier detection of fractures at risk for a healing delay has several potential benefits addressing both the patient’s individual, as well as socioeconomic burden of disease [28]. On the patient side, early interventions ranging from adaptation of the aftercare regime to surgical treatment are possible. As intramedullary nailing usually entails full weight-bearing, potential adaptations can be to reduce weight-bearing in situations with increased interfragmentary motion, while the frequency and intensity of a full weight-bearing state can be addressed in understimulated situations (e.g., climbing stairs or rising from the chair leads to a reaction force much higher than walking in a straight line) [14,15,21]. If the simulation determines that healing cannot be achieved by modulation of the weight-bearing input, the stiffness of the osteosynthesis construct can be adjusted: Dynamization, reverse dynamization, and augmentative plating, as well as exchange nailing, to optimize nail fit are all potential options that could be simulated before surgery and scaled to the individual need [29]. Our preferred method to address long bone non-union in the lower extremity differs depending on the non-union type to be revised [2]. In clinically non-infected cases indicated for surgery, our preferred method in the tibial shaft non-union is exchange nailing, where we aim to increase the nail diameter by at least 2 mm and emphasize a good reaming technique, with stepwise reamer increase by 0.5 mm and final reaming at least 1 mm greater than the aspired nail diameter. In the femur, especially in oligotrophic cases, again exchange nailing is our preferred method. In hypertrophic non-unions, especially in the diametaphyseal region, augmentative plating with local cancellous autograft can be a viable option. The simulation presented in this study can help assess the pathogenesis of a non-union, assisting in clinical decision making. Depending on rotational and axial alignment, as well as potential bone defects, our revision strategy is adapted as individually required [2].

As the simulation workflow is already reasonably fast and could increase in precision and speed pending further adaptations, its application in the future could also be seen as a screening tool, providing added socioeconomic effects on top of the decreased patient burden of disease [2]. As it is run on available clinical information alone, it could potentially also be applied centrally through health or accident insurance organizations, allowing for a large number of patients screened early on.

### Limitations

This study has several underlying limitations. The introduced simulation protocol and the conclusions drawn are based on the case series analysis of two patients, which limits the generalizability of the results due to the low patient number. The presented study serves only as a description of two use cases of the workflow’s clinical applicability. The workflow has been clinically tested in different upper and lower extremity fracture entities during a regular healing course, showing its principal accuracy in union determination [24,25]. In contrast, the current analysis was performed to show its clinical application in challenging healing cases with complex fractures and patients with different pre-existing diseases representing the influence of both mechanical and biological factors on fracture healing, leading to non-union. The determined effects on tissue differentiation cannot be validated experimentally in vivo. Validation can only be given through the observed clinical course in line with the simulation results. The mechanical parameters that the simulation is based on are determined by the implant and weight-bearing condition assumed as per the patient’s body weight. More detailed simulations incorporate musculoskeletal models including muscle and tendon pull based on individualized motion capture to provide a greater degree of detail and with higher resolution in the fracture gap [21]. CT and conventional 2D imaging data were manually matched and aligned, thus introducing potential bias. Another limitation to be mentioned is the possible alterations in gait and weight-bearing after a lower limb fracture. It has to be assumed that patients reduce their weight-bearing when suffering pain. As our second case shows, this has to be taken into consideration in further development and usage of the simulation. However, the combined consideration of both biological and mechanical aspects in the workflow presented here seems to make up for this detail, still providing a simulation result according to the clinical course. 

It can be expected that in the future, by incorporating additional clinical data types in more diverse study setups, also including different and more complex fractures and varying patient conditions, the precision of the model’s predictive capability will certainly increase [37,38,39,40]. This could potentially lead to adapted treatment, as well as aftercare regimes, such as addressing weight-bearing according to the fracture needs [41,42].

## 5. Conclusions

We introduce a workflow that may improve success in predicting fractures at risk for non-union development early during the treatment course in a clinical application scenario. In this use case series, for each patient, the input necessary for simulation is based on available clinical and radiographic information. Further validation in a large non-union cohort is needed to increase the model’s precision, especially in biologically challenging cases, and show its validity as a screening instrument compared to established clinical scores.

## Data Availability

Data supporting this study beyond the presented information are not available.

## Supplementary Materials

The following supporting information can be downloaded at: https://www.mdpi.com/article/10.3390/jcm13133922/s1, Figure S1: Modeled mechanobiological processes of the Ulm tissue-level bone healing model; Figure S2: Steps to apply the simulation model to the investigated cases; Figure S3: Segmentation of the CT data in 3D slicer. Yellow: Bone; Blue: Nail Implant; Violett: Fibula Implant; Figure S4 Meshed investigate femur case with the mentioned healing domain. Depicted in a sliced view from AP; Figure S5 Applied loading condition in the femur case; Figure S6 User interface to track simulation status and analyze the simulation results, provided by OSORA medical GmbH.
