# Supplementary material for: Case Studies of a Simulation Workflow to Improve Bone Healing Assessment in Impending Non-Unions
Source: J Clin Med. 2024 Jul 4;13(13):3922. doi: 10.3390/jcm13133922 (PMC11242056; doi:10.3390/jcm13133922)
Supplement: Supplementary file 1 [file jcm-13-03922-s001.zip › jcm-2907528-supplementary.pdf]

General overview of the used methodology

The bone healing simulation used in this study is based on the Ulm tissue-level bone healing model. After defining the initial tissue distribution in the investigated geometry, it is possible to predict the evolution of woven and lamellar bone, fibrocartilage, and fibrous connective tissue over time [1, 2, 4].

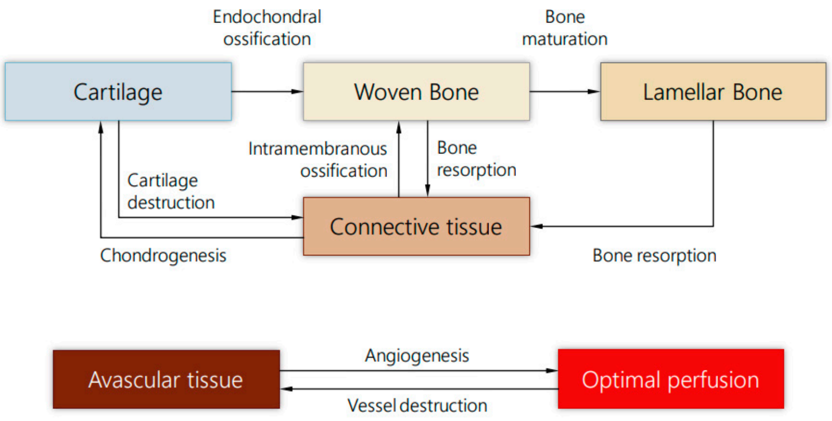

Figure S1 Modeled mechanobiological processes of the Ulm tissue-level bone healing model

The simulation is based on *in vivo* biomechanical observations and the mechano-regulating hypothesis derived from those experiments by Claes and Heigele [5, 6] and takes mechanical and biological stimuli into account. For instance, local tissue differentiation is influenced by adjacent tissues (e.g. bone can only arise close to existing bone surfaces) as well as local vascularization. To simulate the influence of mechanical stimuli, the current mechanical strain at every point in the simulation domain are analyzed via Finite Element Analysis to calculate distortional and dilatational strains. Those strains have been identified to be determining mechanical factors for tissue differentiation and remodeling [4]. This way the presented model predicts the spatio-temporal development of the different tissue types for a chosen time period. Further details regarding the general simulation workflow as well as the mathematical formulation can be found in recent publications [1, 2] and application in Degenhart et al. [3].

Process for the investigated cases

For each of the investigated cases the following procedure was performed:

1. Data collection
2. 3D geometry reconstruction
3. Preprocessing for digital twins
4. Running the fracture healing simulation
5. Deriving outcome measures (i.e. consolidation time points)

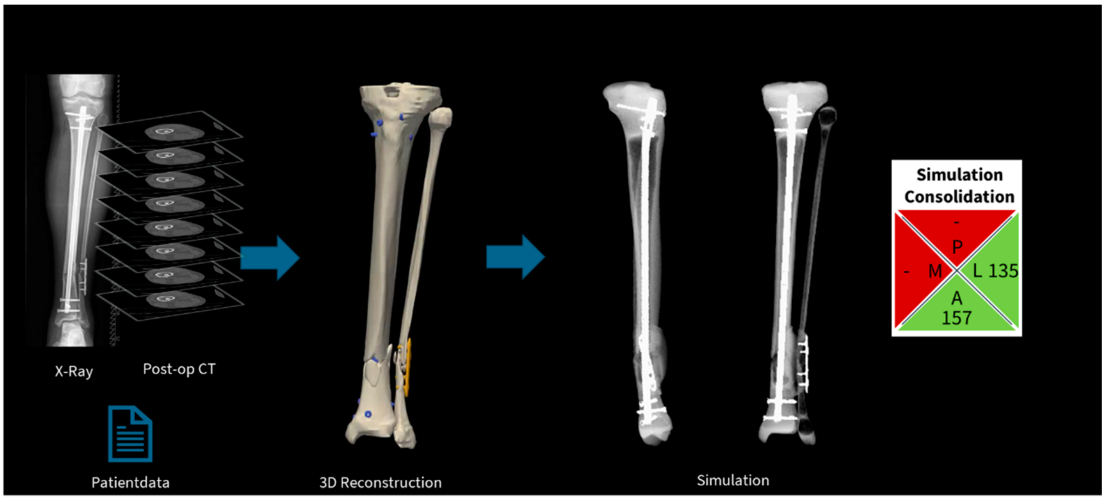

Figure S2: Steps to apply the simulation model to the investigated cases

1. **Data collection**

   To generate the digital twin of a case the following data was collected:
   - Pre- and post-op X-rays
   - Post-op CT scans
   - Weight, height and age of the patient
   - Assumed and recommended weightbearing
   - Comorbidities and other patient habits

2. **3D geometry reconstruction**

   From the provided CT scans a digital geometry was reconstruct from the post-operative situation. For each of the cases, 3D Slicer was used to segment the tibia/fibula or the femur, respectively, as well as the implant from the CT image data. This step enabled the generation of an accurate three-dimensional (3D) geometric representation of the fractured bone. Since the CT scans were not taken immediately after the surgery, but at a later time, we manually realigned the fragments to correspond to the post-operative X-ray scans.

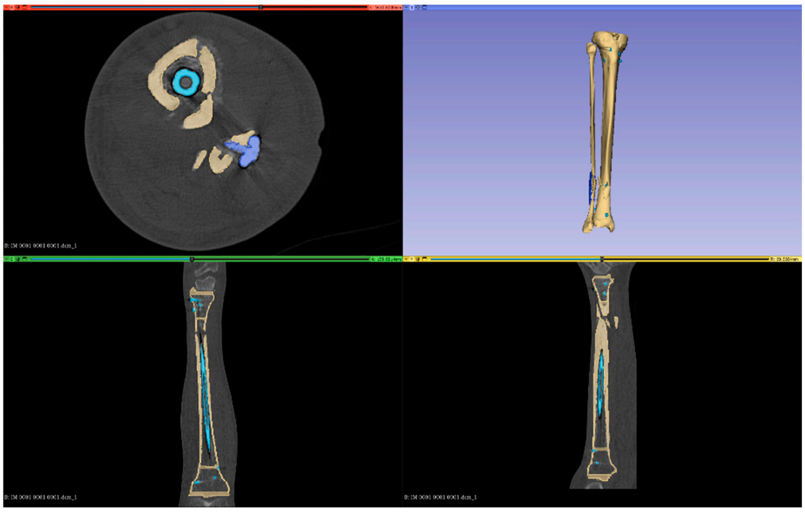

Figure S3: Segmentation of the CT data in 3D slicer. Yellow: Bone; Blue: Nail Implant; Violett: Fibula Implant

3. Preprocessing for digital twins

The fracture area was embedded in a healing domain that covered the entire fractured region. This domain represents the area immediately surrounding the fracture, where tissue formation and differentiation are expected to occur due to mechanical and biological stimuli, based on the tissue differentiation algorithm. The geometries can then be discretized into finite elements using appropriate meshing techniques. For the initial tissue composition, we assumed that the cortical and cancellous bone consisted of 100% fully vascularized lamellar bone, while the remaining tissue (in and near the fracture) was assumed to consist of initially avascular soft tissue.

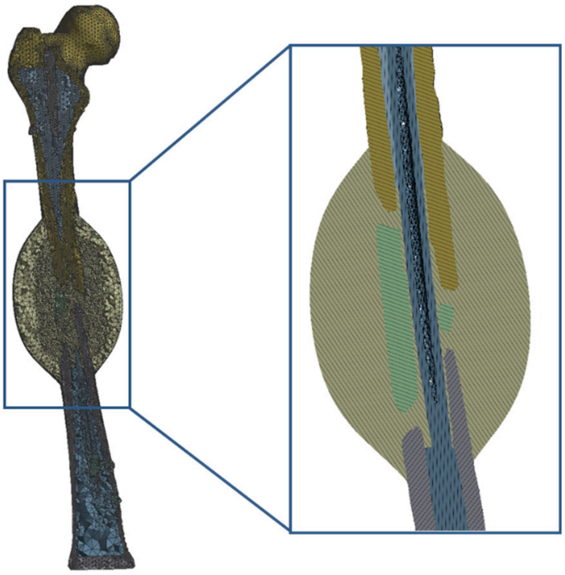

Figure S4 Meshed investigate femur case with the mentioned healing domain. Depicted in a sliced view from AP.

To determine the loading conditions, relevant literature on physiological loading scenarios for femoral and tibia fractures were consulted. These loading conditions were then applied to the FE model to simulate the mechanical response of the fractured bone. In both cases the patients were assumed to bear full weight after surgery. According to our recent study with femur fractures [3] the maximum load occurring during the normal gait cycle was assumed as the representative stimulus. Therefore, the muscle and joint loading conditions, as stated by Heller et al., 2005 [7], were used, which allow to express loading in terms of percentage of patients' bodyweight. The tibia case was loaded as described by Zhao et al. by applying a 55/45% split of the peak load during the gait cycle (2.2 times Bodyweight) on the medial and lateral compartments of the tibial plateau [8].

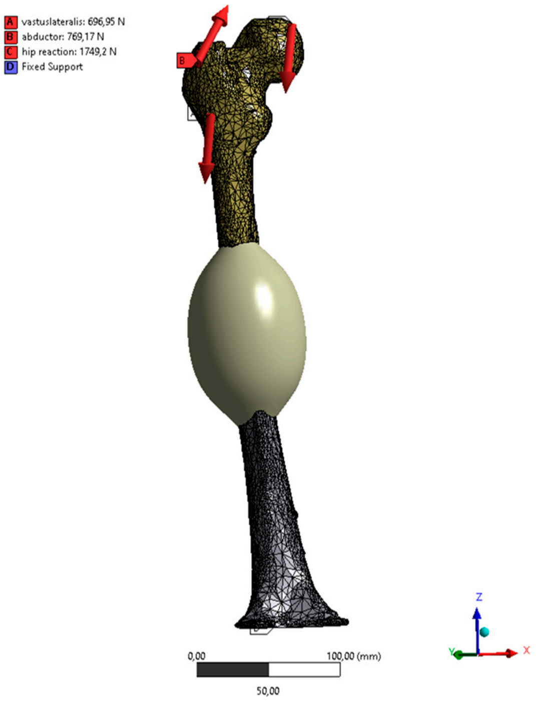

Figure S5 Applied loading condition in the femur case

4. Running the fracture healing simulation

Using the reconstructed geometry and the patient's weight, the simulations were able to be performed within 24 hours of simulation time on a high-performance workstation (AMD Ryzen 5900X, 128 GB RAM). To track simulation and analyze the healing outcome a customized user interface was provided from OSORA medical.

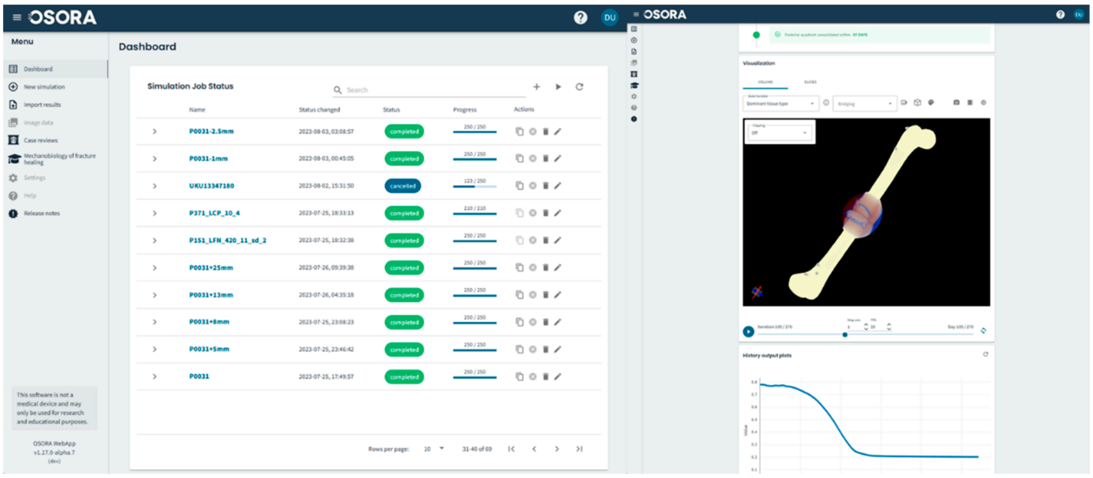

Figure S6 User interface to track simulation status and analyze the simulation results, provided by OSORA medical GmbH

5. Deriving outcome measures

The time of consolidation is derived automatically from the primary simulation results by using a path-search algorithm to detect connections of lamellar bone in all four quadrants of the bone. If the simulation predicts bony bridging in at least three quadrants within the simulated days the case will be classified as "union"; otherwise, the case is classified as a "non-union".

References

1 Niemeyer, F., L. Claes, A. Ignatius, N. Meyers, and U. Simon. "Simulating Lateral Distraction Osteogenesis." *PLoS One* 13, no. 3 (2018): e0194500.

2. Engelhardt, Lucas, Frank Niemeyer, Patrik Christen, Ralph Müller, Kerstin Stock, Michael Blauth, Karsten Urban, Anita Ignatius, and Ulrich Simon. "Simulating Metaphyseal Fracture Healing in the Distal Radius." *Biomechanics* 1, no. 1 (2021): 29-42.

3 Degenhart, Christina, Lucas Engelhardt, Frank Niemeyer, Felix Erne, Benedikt Braun, Florian Gebhard, and Konrad Schütze. "Computer-Based Mechanobiological Fracture Healing Model Predicts Non-Union of Surgically Treated Diaphyseal Femur Fractures." *Journal of Clinical Medicine* 12, no. 10 (2023): 3461.

4 Simon, U., P. Augat, M. Utz, and L. Claes. "A Numerical Model of the Fracture Healing Process That Describes Tissue Development and Revascularisation." *Comput Methods Biomech Biomed Engin* 14, no. 1 (2011): 79-93.

5 Pauwels, Friedrich. "Eine Neue Theorie Über Den Einfluß Mechanischer Reize Auf Die Differenzierung Der Stützgewebe." *Zeitschrift für Anatomie und Entwicklungsgeschichte* 121, no. 6 (1960): 478-515.

6 Claes, L. E., and C. A. Heigele. "Magnitudes of Local Stress and Strain Along Bony Surfaces Predict the Course and Type of Fracture Healing." *J Biomech* 32, no. 3 (1999): 255-66.

7 Heller, M. O., G. Bergmann, J. P. Kassi, L. Claes, N. P. Haas, and G. N. Duda. "Determination of Muscle Loading at the Hip Joint for Use in Pre-Clinical Testing." *J Biomech* 38, no. 5 (2005): 1155-63.

8 Zhao, D., S. A. Banks, D. D. D'Lima, C. W. Colwell, Jr., and B. J. Fregly. "In Vivo Medial and Lateral Tibial Loads During Dynamic and High Flexion Activities." *J Orthop Res* 25, no. 5 (2007): 593-602.
